# Assessing the Suitability of ChatGPT and DeepSeek AI for Patient Education on Common Rheumatological Disorders

**DOI:** 10.7759/cureus.90600

**Published:** 2025-08-20

**Authors:** Amruth A Alluri, Zakiya Khan, V Krithika, Maira Jalil, Gayathri Dantu, Kalyan Kumar Reddy Annapureddy, Shreya Deoghare

**Affiliations:** 1 Internal Medicine, American University of the Caribbean School of Medicine, Cupecoy, SXM; 2 Internal Medicine, Thanjavur Medical College, Thanjavur, IND; 3 Critical Care, Sri Ramachandra Institute of Higher Education and Research, Chennai, IND; 4 Internal Medicine, University of Debrecen, Faculty of Medicine, Debrecen, HUN; 5 Internal Medicine, Government Medical College, Mahabubnagar, IND; 6 Internal Medicine, Sri Venkateswara Medical College, Tirupati, IND; 7 Dermatology, NKP Salve Institute of Medical Sciences, Nagpur, IND; 8 Dermatology, Alexis Multi-speciality Hospital, Nagpur, IND

**Keywords:** chatgpt, deepseekai, dermatomyositis, systemic lupus erythematosus (sle), systemic sclerosis

## Abstract

Introduction

Systemic lupus erythematosus (SLE), systemic sclerosis, and dermatomyositis are among the most prevalent rheumatological conditions. Using artificial intelligence tools (AI) like ChatGPT and Deepseek AI in health care can help in providing personalized patient education, resulting in improved health care literacy and treatment adherence.

Aim

To assess and compare ChatGPT and DeepSeek AI's effectiveness in generating understandable, accurate, and reliable patient education guide for three rheumatological conditions: systemic lupus erythematosus, systemic sclerosis, and dermatomyositis.

Methodology

ChatGPT 4.0 and DeepSeek AI were asked to write a patient education guide for “systemic lupus erythematosus", "systemic sclerosis," and " dermatomyositis". These materials were assessed using validated readability scores (Flesch-Kincaid, grade level, ease score), linguistic complexity analysis (average syllables per word, words per sentence), and similarity metrics to standard rheumatological resources. Finally, the reliability was rated using " discern score, a structured evaluation framework formed based on evidence-based guidelines from the British Society of Rheumatology and the American College of Rheumatology.

Results

There was no significant difference in the word count (p=0.775), sentence count (p=0.802), average word per sentence (p=0.349), average syllables per sentence(p=0.101), grade level (p=0.193), similarity% (p=0.481), reliability score (p=0.742), and ease score (p=0.097) between ChatGPT and Deepseek AI.

Conclusions

Both ChatGPT and DeepSeek AI offer promising avenues for augmenting patient education in rheumatology, but due to the limitations, their output should be used as a complement - not a replacement - for verified, expert-reviewed educational materials.

## Introduction

Systemic lupus erythematosus (SLE), systemic sclerosis, and dermatomyositis are among the most prevalent rheumatological and connective tissue diseases. SLE is a chronic autoimmune disease that affects multiple systems and has a relapsing and remitting course [1]. Systemic sclerosis is an immune-mediated disease that is defined by vasculopathy and fibrosis of the skin and internal organs [2]. Skeletal muscle inflammation, symmetrical proximal myopathy, and cutaneous symptoms are the hallmarks of dermatomyositis, an idiopathic acute inflammatory disease [3]. Patient education intends to promote participation in treatment decision-making by offering sufficient clinical information to improve perception of diseases. Patients benefit from this process by gaining insights about preventative care, basic medical issues, proper medication usage, and at-home treatment for injuries and illnesses. This encourages healthy lifestyles, patient compliance, and satisfaction with care. Additionally, patient education has contributed to fewer negative hospital experiences, such as decreased anxiety and pain levels [4].

The use of artificial intelligence (AI) for patient education has several advantages. AI can adjust to the distinctive needs of every patient, allowing for a more focused and customized educational approach that improves decision-making and treatment effectiveness, as judged by readability, reliability, and similarity to standard references, as later outlined in the methodology. It makes patient education more engaging by incorporating interactive components and encouraging patients to actively participate in the learning process by asking questions and receiving real-time feedback. AI mobile and web platforms provide patients prompt access to educational information, removing geographical boundaries [5]. However, AI may also propagate false information on an unprecedented scale. According to one study, an AI model could generate 102 blog posts containing over 17,000 words of false content [6]. Furthermore, the digital divide creates accessibility barriers since people from different socioeconomic backgrounds might not be able to access the required technology [7]. ChatGPT is an advanced linguistic model that uses deep learning methods to generate human-like responses to natural language inputs [8]. Natural language processing (NLP) and deep learning technologies form the foundation of the artificial intelligence platform DeepSeek [9].

By improving communication and education, AI platforms such as ChatGPT and DeepSeek can be useful resources in patient consultation for common rheumatological disorders such as lupus, scleroderma, or dermatomyositis. For improving health literacy and treatment adherence, ChatGPT can help patients better understand complicated medical information. It can provide real-time answers to frequently asked queries and emotional support, enhancing the advice of clinicians. By evaluating each patient's specific predicament, DeepSeek, with its sophisticated reasoning and multi-modal capabilities, can help with treatment selection. These AI tools can facilitate shared decision-making, expedite consultations, and enhance patient outcomes.

The aim of this study is to assess and compare ChatGPT and DeepSeekAI's effectiveness in generating understandable, accurate, and reliable patient education guide for three rheumatological conditions: systemic lupus erythematosus, systemic sclerosis, and dermatomyositis.

## Materials and methods

A cross-sectional study was conducted over a period of one week, from March 20 to March 27, 2025. Since there were no human participants involved in the study, ethics committee approval was not needed.

Three common rheumatological conditions such as systemic lupus erythematosus, systemic sclerosis, and dermatomyositis were selected. Two Artificial Intelligence tools were selected for the generation of patient education brochures: ChatGPT (OpenAI, GPT-4, March 2025 version) and DeepSeek AI (March 2025 version) [10,11]. For each condition, a standard prompt was given to both tools: “Write a patient education guide for (Systemic Lupus Erythematosus/ Systemic Sclerosis/ Dermatomyositis).” The responses generated were collected in a Microsoft Word Document without any modifications. The responses were graded using the following parameters:

1. Readability was assessed using the Flesch-Kincaid calculator, which provided metrics including word count, sentence count, average words per sentence, grade level, ease-of-understanding score, and overall readability [12]. A higher ease score indicated that the content was easier to read. The grade levels were interpreted as follows: a grade level between 6 and 8 was considered appropriate for middle school and suitable for most patient education materials; a grade level between 9 and 11 indicated more complex text that might be moderately challenging for general readers; and a grade level of 12 or above signified high complexity, which could be too difficult for the average patient to understand.

2. Similarity was assessed using Quillbot’s plagiarism checker, which evaluated the percentage of overlap with existing online sources [13]. This analysis helped determine the originality of the AI-generated content.

3. Reliability was assessed using a modified DISCERN score, a validated instrument designed to evaluate the quality and trustworthiness of written health information by examining factors such as clarity of aims, use of evidence-based sources, and balance of content [14]. Five parameters were evaluated for each AI-generated document, including whether the content stated its aims clearly, used trustworthy and referenced sources, presented information in a balanced and unbiased way, offered further resources or guidance, and acknowledged any uncertainties or gaps in information. Each parameter received a score of 1 if present or 0 if absent, resulting in a total possible score between 0 and 5. The scores were interpreted as follows: scores between 0 and 1 indicated poor reliability, scores between 2 and 3 indicated moderate reliability, and scores between 4 and 5 reflected high reliability. The modified version used in this study employed a five-item rubric, with each item scored from 1 (lowest) to 5 (highest), covering: (1) clarity of objectives, (2) relevance and completeness of information, (3) transparency of information sources, (4) balance and lack of bias, and (5) inclusion of areas of uncertainty.

All data was exported to a Microsoft Excel (Microsoft Corp, Redmond, NY) sheet. Statistical analysis was done using R version 4.3.2 (R Foundation for Statistical Computing, Vienna, Austria). To compare the responses generated by ChatGPT and DeepSeek AI, an unpaired T-test was used. A p-value of less than 0.05 was considered statistically significant. The relationship between ease score and reliability score was studied using the Pearson’s coefficient of correlation.

## Results

**Figure 1:**
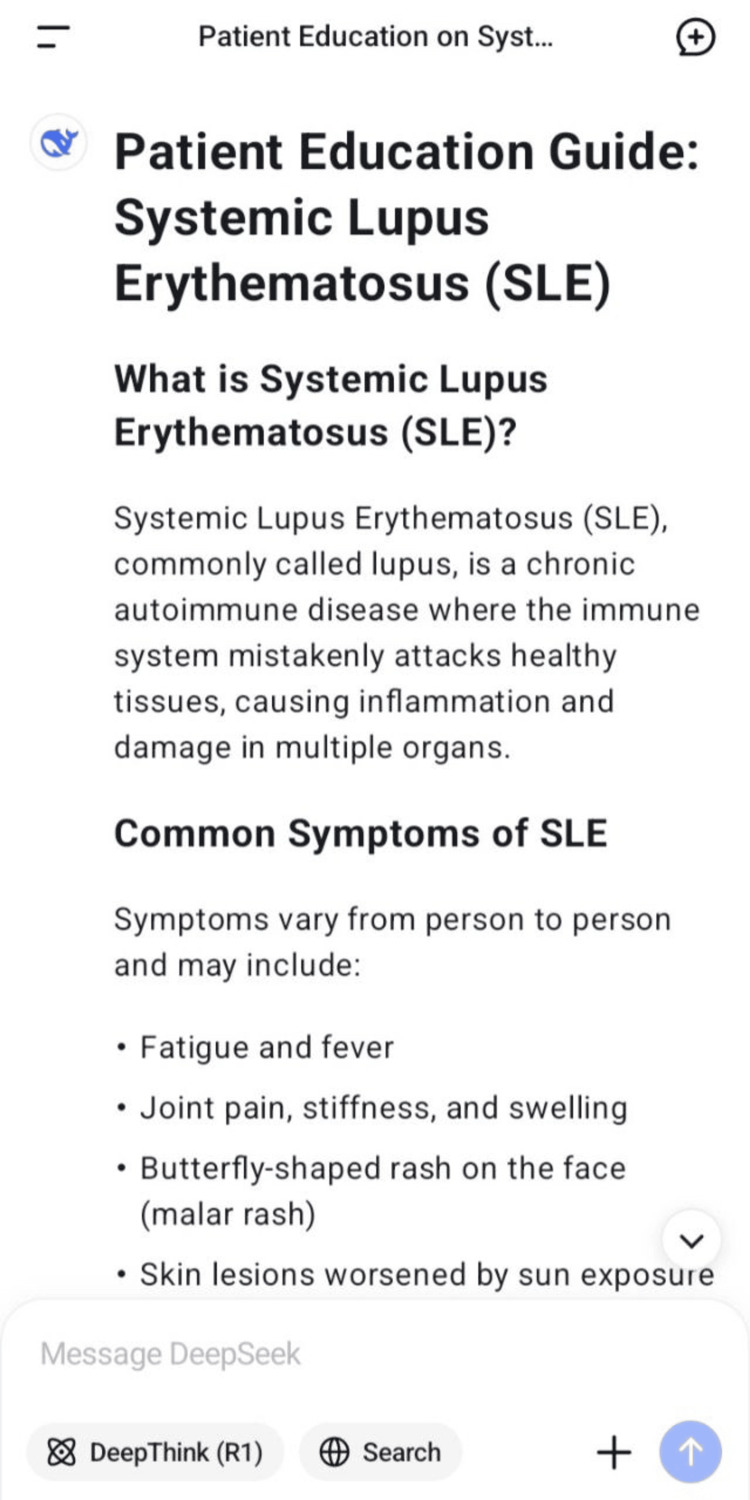
Response generated by DeepSeek AI

**Figure 2:**
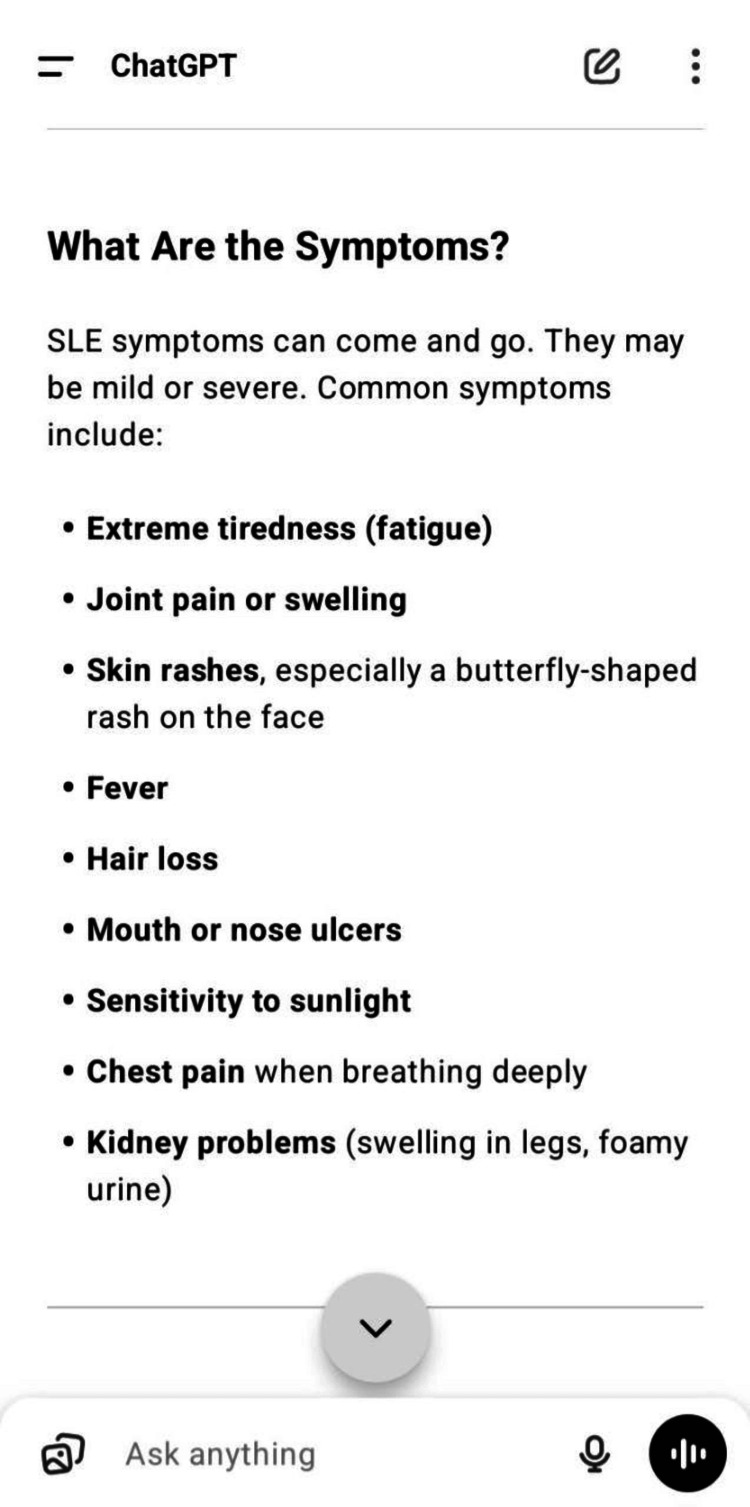
Response generated by ChatGPT

ChatGPT and DeepSeekAI were used to generate brochures on patient education for systemic lupus erythematosus, systemic sclerosis, and dermatomyositis. Figure 1 illustrates the response generated by DeepSeek AI. Figure 2 represents the response generated by ChatGPT. Table 1 shows that there was no significant difference in the word count (p=0.775), sentence count (p=0.802), average word per sentence (p=0.349), average syllables per sentence (p=0.101), grade level (p=0.193), similarity% (p=0.481), reliability score (p=0.742) and ease score (p=0.097) between ChatGPT and Deepseek AI.

**Table 1:** Characteristics of responses generated by ChatGPT and DeepSeekAI

	ChatGPT		DeepSeekAI		t-value	P value
	Mean	Standard Deviation	Mean	Standard Deviation		
Words	548.67	193.74	507.67	127.69	0.306	0.775
Sentences	77.67	29.14	83.00	18.33	-0.268	0.802
Average Words per Sentence	7.37	1.96	6.13	0.46	1.061	0.349
Average Syllables per Word	1.97	0.06	2.07	0.06	-2.121	0.101
Grade Level	10.50	0.46	11.20	0.62	-1.565	0.193
Ease Score	33.00	3.50	25.77	4.64	2.156	0.097
Similarity %	46.13	7.01	36.43	20.50	0.775	0.481
Reliability Score	3.33	1.53	3.67	0.58	-0.354	0.742
P-values <0.05 are considered statistically significant.						

Independent sample t-test has been used to compare the means between ChatGPT and DeepSeek AI. Normality of the variables was assessed using Shapiro-Wilk test and equality of variances was tested using Levene’s test. Based on the P values obtained in Table 1, there is no statistically significant difference between any of the responses generated by the two AI tools.

1. Word Count: ChatGPT generated slightly longer responses (M=548.67, SD=193.74) compared to DeepSeek AI (M=507.67, SD=127.69), indicating a tendency for ChatGPT to provide more verbose answers. However, this difference was not statistically significant (p=0.775).

2. Sentence Count: DeepSeek AI produced marginally more sentences per response (M=83.00, SD=18.33) than ChatGPT (M=77.67, SD=29.14). This suggests that DeepSeek AI may segment its information into shorter chunks, though the difference was minimal (p=0.802).

3. Average Words per Sentence: ChatGPT responses were more syntactically complex, with longer sentences on average (M=7.37 words, SD=1.96) than those from DeepSeek AI (M=6.13 words, SD=0.46). Although not statistically significant (p=0.349), this indicates that ChatGPT may use more elaborate sentence structures.

4. Average Syllables per Word: DeepSeek AI exhibited slightly higher lexical complexity, using words with more syllables on average (M=2.07, SD=0.06) than ChatGPT (M=1.97, SD=0.06). While the difference approached significance (p=0.101), it may suggest a preference for more complex vocabulary by DeepSeek AI.

5. Grade Level: Responses from DeepSeek AI scored higher on the grade level metric (M=11.20, SD=0.62) than those from ChatGPT (M=10.50, SD=0.46), indicating a slightly more advanced reading level. However, this difference was not significant (p=0.193), and both models produced content at a senior high school or early college level.

6. Ease Score (Flesch Reading Ease): ChatGPT responses were easier to read (M=33.00, SD=3.50) compared to DeepSeek AI (M=25.77, SD=4.64). Although not statistically significant (p=0.097), this metric aligns with prior results, suggesting that DeepSeek AI produces denser, more formal language that may be harder for general readers to digest.

7. Similarity Percentage: ChatGPT showed a higher average similarity percentage (M=46.13, SD=7.01) compared to DeepSeek AI (M=36.43, SD=20.50), indicating potentially more consistent phrasing or closer adherence to input structure. However, due to high variability in DeepSeekAI outputs, this difference was not significant (p=0.481).

8. Reliability Score: Reliability scores, possibly reflecting factual correctness or coherence (scoring scale not specified), were comparable between the two models: ChatGPT (M=3.33, SD=1.53) and DeepSeek AI (M=3.67, SD=0.58), with no significant difference (p=0.742).

## Discussion

A cross-sectional study conducted to compare responses generated by two AI tools, ChatGPT and DeepSeek AI, for brochures on patient education for systemic lupus erythematosus, systemic sclerosis, and dermatomyositis, revealed no statistically significant differences between the two tools in terms of word count, readability (ease score), grade level, similarity percentage, and reliability scores. Specifically, the readability and reliability of content generated by both AI platforms were similar, with no significant discrepancies noted.

Artificial Intelligence has emerged as an innovative method to enhance patient education, particularly in the dissemination of concise and comprehensible information tailored for broad audiences [15-17]. Effective patient education materials are critical, as clear, easily understood content can significantly improve patient compliance and health outcomes by ensuring patients adequately understand their conditions and treatment options [14]. Previous studies underscore that patient education materials should ideally be simple enough to be understood by individuals with a high school education [18]. Our study showed that the average ease score was 33.00 for ChatGPT and 25.77 for DeepSeek AI, indicating that both AI tools produced content slightly challenging for individuals below a high school education level, suggesting room for improvement.

AI-generated content often exhibits considerable similarity due to reliance on extensive pre-existing training datasets, raising concerns regarding potential plagiarism [19,20]. Plagiarism in medical literature is detrimental as it undermines the originality and integrity of scientific communication, potentially propagating inaccuracies and hindering academic credibility [21]. In this study, the similarity percentage averaged 46.13% for ChatGPT and 36.43% for DeepSeekAI, both relatively high, underscoring the need for cautious interpretation and additional originality checks in medical content generated by AI tools. Similar studies have highlighted comparable concerns, advocating for stringent plagiarism checks and improved AI training protocols to mitigate these issues.

Reliability in patient education material is crucial and is typically assessed using tools like the modified DISCERN score, which evaluates the accuracy, evidence base, and reliability of health information [22]. This scoring system assists patients and providers in determining the trustworthiness of online health information. Our analysis revealed an average modified DISCERN score of 3.33 for ChatGPT and 3.67 for DeepSeek AI, indicating moderate reliability. Previous research using DISCERN scores also reports similar moderate reliability levels for AI-generated educational content, reinforcing the necessity for human oversight to ensure clinical accuracy and appropriateness [23,24].

Limitations

Several limitations of our study warrant mention. Firstly, the evaluation included only two AI tools, limiting the generalizability of our findings. Future research should include a broader range of AI platforms to establish comprehensive conclusions regarding their suitability in patient education. Additionally, the study evaluated only three specific rheumatological disorders, necessitating broader analyses encompassing diverse diseases to better understand the general applicability of these AI tools. Moreover, the inherent variability in AI-generated outputs and the absence of standardized parameter settings may have influenced the results, potentially affecting consistency across trials. Our use of an older version of ChatGPT may have influenced content currency and accuracy, emphasizing the need for repeated studies with updated AI versions to ensure contemporary relevance [25]. Reproducibility was also not fully assessed, and potential subjectivity in scoring (e.g., interpretation of DISCERN criteria) may have introduced inter-rater variability, underscoring the importance of including inter-rater reliability measures in future research.

## Conclusions

Based on the evaluation, the study concludes that ChatGPT produces content that is more readable, as indicated by lower grade levels and higher ease scores, particularly for systemic lupus erythematosus and systemic sclerosis but not to an extent that is statistically significant. Its output demonstrates a greater similarity to reference information for these two conditions, suggesting better alignment with established knowledge.

These findings emphasize that neither AI tool consistently outperforms the other across all evaluated parameters and rheumatological conditions. These AI tools could serve as valuable supplementary resources for patients seeking accessible and understandable medical information. However, limitations such as potential inaccuracies or oversimplifications are also highlighted, pointing out the need for professional oversight in AI-assisted patient education, while careful evaluation and tailoring are essential to ensure accuracy and accessibility.

Furthermore, this comparative analysis provides a clear scientific rationale for selecting the AI system that best matches the intended purpose of generating patient education material. For instance, given ChatGPT’s strengths in readability and alignment with reference information, it may be better suited for producing accessible patient-facing content, whereas DeepSeek’s technical depth may be more appropriate for clinician-oriented or highly specialized educational resources. By clarifying the distinct capabilities of each system, the study offers actionable insights for matching the AI tool to the specific communication and educational needs of the target audience.
